# *‘Let the System Do Its Job and Families Handle the Rest’?* Protective and Risk Factors Contributing to Pandemic Crisis Parental Burnout

**DOI:** 10.3390/jcm14020617

**Published:** 2025-01-18

**Authors:** Roman Ryszard Szałachowski, Wioletta Tuszyńska-Bogucka, Jacek Bogucki

**Affiliations:** 1 Faculty of Social Sciences, University of Szczecin, 71-017 Szczecin, Poland; roman.szalachowski@usz.edu.pl; 2 Faculty of Human Sciences, University of Economics and Innovation in Lublin, 20-209 Lublin, Poland; 3 Department of Medicine, John Paul II Catholic University of Lublin, 20-950 Lublin, Poland; jacek.bogucki@kul.pl

**Keywords:** parental burnout, family crisis, balance theory of risk and support factors, COVID-19, pandemic stress, structural equation method

## Abstract

**Background:** The work main purposes were to identify the sources of problems and demands causing parental burnout and to specify the resources/support factors during the COVID-19 pandemic. The study was based on the Balance Theory of Risk and Support/Resource Factors (BR^2^ Model) by Mikolajczak and Roskam. **Methods:** The study explored the predictive value of socio-economic variables, religiosity, the meaning of life, positivity, perceived social support, family functionality, and balance between risks and resources in parental burnout using the structural equation modelling method on a sample of 337 parents. **Results:** The presence of children’s learning difficulties and behavioural problems are the most important risk factors and aggravate parental burnout, and the presence of a meaning of life, support coming from the family, family affection, and relationship lengths are the main protective resources, allowing parental burnout to decrease during the pandemic crisis. **Conclusions:** The findings are instructive for both theory and practice. The study successfully operationalised the BR^2^ model—the model obtained from the path analysis fits well, confirms the structure of parental burnout theory, and demonstrates the appropriateness of the application of BR^2^ theory in crisis conditions. The most effective way to help parents in a crisis situation is (in addition to psychological support) the effective provision of specialist help for children, resulting in a reduced risk of an unfavourable balance between demands and family resources. The family- or parent-oriented interventions that address professional help in problems with children can be the most effective at reducing the negative consequences of a pandemic on children and their parents. The COVID-19 pandemic has shown the importance of investing in healthcare infrastructures.

## 1. Introduction

The COVID-19 pandemic seems to have had far-reaching and long-lasting global effects [1,2,3] including on the functioning of parental roles, causing stress for parents. Although the term parental burnout is not a new construct (it appeared in the literature as early as the 1980s), it takes on special significance today, as the 21st century seems to make special demands in the exploration of this area due to the numerous problems of parental functioning [4,5]. Fortunately, there are emerging successful attempts to objectify its measurement by means of standardised diagnostic tools, successfully adapted in many countries, facilitating the exploration of this area.

This work has the following goals: (1) to identify the sources of problems, and demands, on the mental health of parents resulting from parental burnout in times after crisis, thus facilitating the offering of comprehensive care and support; (2) to specify risk factors and resources in specific situations; (3) to implement the proposal of the authors of the BR^2^ model in order to adapt the model to other cultures.

### 1.1. Parental Burnout in Theoretical Context

#### 1.1.1. Structure of the Syndrome

Since 2017, Roskam and Mikolajczak et al. [6,7,8,9] have provided evidence in favour of the existence of parental burnout, which is defined by them as a four-dimensional set of well-being parameters that result from prolonged exposure to chronic parenting stress, which may result in a parent’s sense of incompetency in their role as a parent:

(1) Emotional exhaustion in parental role. The first element of parental burnout is the appearance of symptoms indicative of parental exhaustion, such as headaches, difficulty sleeping, susceptibility to illness, food aversion, and difficult emotions, such as frustration, resentment, and irritation. Both the fatigue felt by the parent at any given time and the general exhaustion of their role cause the parent not to feel any enjoyment, or to feel much less enjoyment, when spending time with their child. The parent may experience significant changes in mood and respond inappropriately to the child’s needs.
(2) Contrast in parental self. This is followed by psychological problems—a contrast in parental self, together with the consequences—such as a sense of futility and of not being good enough, which usually results in an increased lack of sense of purpose. A parent who has a sense of futility feels that their actions cannot have the desired effect, causing the parent to feel resigned and discouraged. The efforts in the face of social demands and one’s own expectations appear inadequate to the parent, and, as a result, the parent’s sense of self-efficacy decreases.
(3) Emotional distancing. Lack of self-confidence and being at the end of one’s tether leads the parent to emotionally distance themselves. Both the reduction in emotional resources and the creation of mental emotional distancing, as well as overwhelming fatigue, cause the caregiver’s emotional responses to the various situations in which they are involved to become inadequate. The parent performs their duties in an automatic, instrumental way, focusing on the functional aspects and not on the emotional needs of the child. By doing so, they do not allow closer emotional bonds with their children to develop. While still trying to fulfil their care and household responsibilities to the best of their ability, they neglect the area of parenting that involves building relationships and support. Previously active interactions are reduced to the bare minimum.
(4) Feelings of being fed up with the parental role. Due to fatigue and distancing, a parent may find themselves in a situation where fatigue is in no way offset by closeness and bonding with the child. Their role, or rather the performance of it, becomes unpleasant. There is a lack of involvement, which can lead to parental neglect of the child and the need for the parent to escape [6,7,8,9,10,11,12,13].

#### 1.1.2. BR^2^ Model

**Figure 1:**
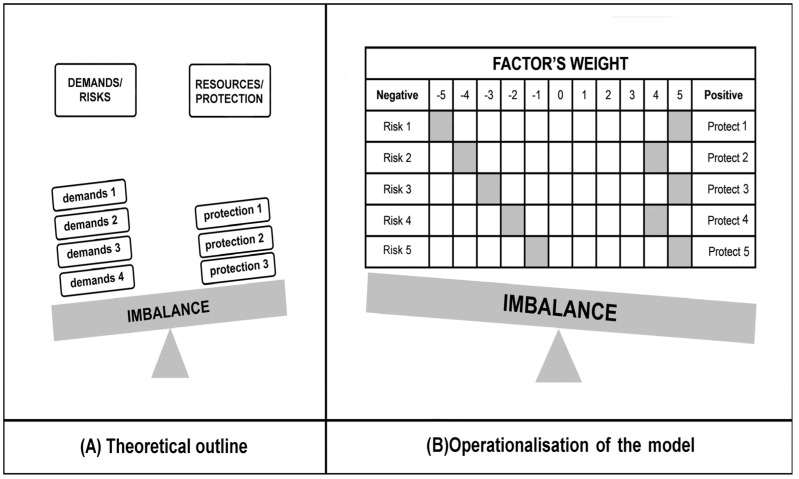
The BR^2^ model of risk/protection factors of parental burnout: (**A**) an example of the predominance of risk factors) and the proposed operationalisation; (**B**) a hypothetical example of the predominance of protection factors [14].

The Balance Theory of Risk and Support/Resource Factors (BR^2^ Model) by Mikolajczak and Roskam [14] is currently the dominant concept used to explain individual differences in parental burnout. Risk factors for parental burnout are considered to be those that contribute to the build-up and persistence of chronic parental stress. In turn, factors that reduce the risk of parental burnout are all those resources that make it possible to reduce it (Figure 1). BR^2^ suggests that some parents may be at greater risk of parental burnout if there is a chronic imbalance between parental demands (i.e., risk factors) and resources, i.e., protective factors [14]. According to Mikolajczak and Roskam, however, not all risk factors are equal, and some risk factors have a greater impact on parents’ feelings regarding emotional exhaustion in terms of the parental role and burnout. According to BR^2^ theory, parents who have greater demands/burdens in relation to resources may have heightened feelings of parenting stress. Continuous exposure to an imbalance between demands and resources may therefore increase the risk of parental burnout [14,15]. The authors of the model confirm that the BR^2^ as an instrument reliably measures parents’ balance between risks (factors that increase parental stress) and resources (factors that alleviate parental stress) and that there is a strong linear relationship between BR^2^ score and parental burnout [14].

#### 1.1.3. Parenting During Pandemic in Research: Risk and Protective Factors

Many of the COVID-19 pandemic conditions were consistent with factors that have been shown to be predictive of parental stress. The literature review has provided an empirical basis to acknowledge that parents face serious stresses and burdens resulting from pandemics:

(1) Psychopathological problems, e.g., severity of anxiety of various types [16,17,18,19,20], depression [19,21,22,23,24,25], stress, and similar symptoms [23,24,25].
(2) A deficit of external resources. This may mainly concern the lack of contact with loved ones–‘to be left without any little help of friends’ [26], problems with access to medical care [27], psychological care [28], or an inability to participate in important events, customs, or rituals, e.g., religious practices [29,30,31,32,33].
(3) Restructuring of parental time and responsibilities [34,35].
(4) Competing demands between work and parental responsibilities at unprecedented levels [34].
(5) The accumulation of serious problems such as unemployment, poverty, addiction, or domestic violence, as well as children’s problems [36,37,38].

However, it is also important to identify and explore protective resources/factors that may prevent parental burnout. Examples of protective factors, found in past studies (pre- and pandemic period), include:

(1) Positive emotionality in the parenting dyad [14,39,40,41,42,43].
(2) Systemic family resources, such as external support from family, friends, or other sources, positive parenting practises, and leisure time (i.e. the developmental spending of leisure time) [14,44,45,46], as well as relationship satisfaction, understood as a positive subjective sense of relationship quality [47,48,49].
(3) Positivity, i.e., keeping a positive attitude and hope as important emotional protective factors that help individuals find resilience in the face of disaster [39,50,51,52,53].
(4) Religiosity, and spirituality, as a resource [54,55,56,57,58,59,60,61,62,63] that is manifested not only in religious practises but also in the search for meaning in life [64,65,66,67,68,69]. It is difficult to define the word spirituality precisely, as it is becoming increasingly separated from religious traditions [70], and it can be observed that the word refers to the deeper values and views on life that people want to pursue. It denotes the search for something that can help a person reach the fullest potential of their life, find answers to their fundamental questions and the meaning of their life, and to realise it to its full potential [71,72,73]. Spirituality, in this view, refers to the dynamic dimension of human life, which refers to the way people experience purpose and meaning in life [74].

## 2. Materials and Methods

### 2.1. Study Design

**Figure 2:**
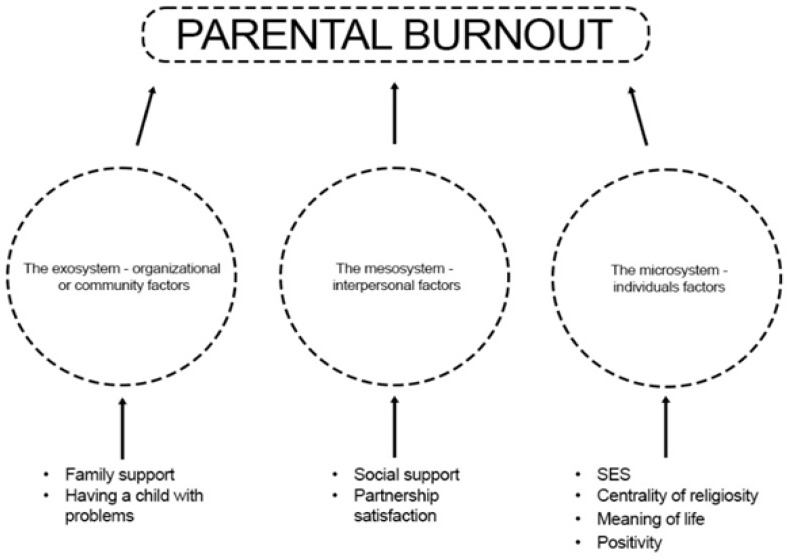
Research project–variables model.

There is the huge need for dynamic multivariate models to understand the causation of parenting stress, because parenting has been shown to be a both complex and stressful activity [8,10,12]. The classification of variables could be derived from human ecological theory, which was adapted by Abidin in the causation model of parenting stress [75] to understand how individuals interact with each other and with their environment [76]. It divides factors into microsystem (individual), mesosystem (interpersonal), and exosystem (organisational or community). Based on the analysis of the literature, a research project was designed and launched based on the BR^2^ concept, that takes into account those psychological and sociodemographic parameters which, as described in the literature, have been shown to be important from the point of view of mitigating or exacerbating parental burnout (Figure 2).

The first predictive hypothesis (H1) assumes an influence by exogenous/independent variables (micro-, meso-, and exosystem variables) on the reality represented by the latent endogenous/dependent variable (parental burnout level in the COVID-19 pandemic). An additional hypothesis (H2) is that the pandemic triggers an imbalance situation in favour of burden. The project is grounded in the BR^2^ model theory and uses both its understanding of the underlying concepts and methodology.

### 2.2. Data Sources

Parental burnout was assessed using the Parental Burnout Assessment (PBA) by Roskam et al. [7,9], adapted in Poland by Szczygieł et al. [77]. It is a self-report questionnaire with 23 items, consisting of 4 subscales: (1) emotional exhaustion in parental role (9 items; e.g., ‘I feel completely run down by my role as a parent’); (2) contrast in parental self (6 items; e.g., ‘I tell myself I’m no longer the parent I used to be’); (3) emotional distancing (3 items; e.g., ‘I am no longer able to show my children that I love them)’; and (4) feelings of being fed up with the parental role (5 items; e.g., ‘I do not enjoy being with my children’). Each item was rated on a 7-point Likert scale from ‘never’ to ‘every day’. A total parental burnout score was obtained by summing each item, with high total scores indicating greater parental burnout. The α—Cronbach reliability coefficient value for the subscales remained in the range of 0.88 to 0.95 and 0.97 for the overall parental burnout scale [8,77].

The Centrality of Religiosity Scale (CRS) by Huber [78], in the Polish adaptation by Zarzycka [79], is a measure of the centrality, importance, or salience of religious meanings in personality. It consists of five subscales: (1) interest in religious issues—the frequency and significance of cognitive confrontation with religious content, regardless of the aspect of their personal acceptance; (2) religious beliefs—the degree of subjectively assessed probability of the existence of a transcendent reality and the intensity of openness to various forms of transcendence; (3) prayer—the frequency of contact with a transcendent reality and the subjective significance of this contact; (4) religious experience—the frequency with which transcendence becomes part of human experience and the degree to which the transcendent world of religious significance is individually confirmed through the meaning of communication and action; and (5) cult—the frequency and subjective significance of a person’s participation in religious services (the dimension of public practice). The overall result is the sum of the subscale scores and is a measure of the Centrality of religiosity in an individual’s personality. The scale consists of 15 items with a Likert scale, and respondents reply by selecting from 5 to 8 possible answers. In every instance, responses are transposed onto a 5-point scale (the higher the score, the greater the significance/frequency of behaviour). The reliability of the scale was estimated using α—Cronbach coefficient and is as follows: 0.82 ≤ α ≤ 0.90. The values of intercorrelation between items and the score in individual subscales indicate the accuracy of a separate theoretical construct, and the subscales can be considered homogeneous [79].

The Meaning of Life Questionnaire (MLQ) by Steger et al. [73] was used to measure the Sense of meaning of life, measuring declared Meaning of life in the present time (subscale Presence of meaning of life), and in the future time perspective (subscale Search for meaning of life). The Polish adaptation was carried out by Kossakowska et al. [80]. In the 10-item questionnaire, the answer ‘1’ denotes ‘strongly disagree’ and ‘7’ indicates ‘strongly agree’. As the authors of the Polish adaptation indicate, the reliability of the questionnaire is satisfactory, and the issues of accuracy require further research and discussion, particularly in an intercultural context. Reliability, as measured by the α—Cronbach coefficient, was 0.87 for the MLQ—P subscale, and 0.81 for the MLQ—S [80].

The study used the Positivity Scale (PS) by Caprara et al. [81], as adapted by Łaguna et al. [82], which is designed to measure positivity, defined as a pervasive mode of appraising, viewing, and perceiving life from a positive stance. It is a variable that combines three components: self-esteem, optimism, and life satisfaction. The PS consists of 8 items (rated on a 5-point scale) covering individuals’ perceptions of being worthy of value, individuals’ positive expectations about the future, and individuals’ satisfaction with their lives. The answer ‘1’ denotes ‘strongly disagree’ and ‘5’ indicates ‘strongly agree’. The higher the score obtained, the higher the level of positivity. The α—Cronbach coefficient of the tool is 0.77–0.84 [82].

Despite Yaphe’s harsh assessment of the FAPGAR [83], many authors emphasised its usefulness and satisfactory psychometric properties, especially in screening studies of family functionality. These included works from the last century [84,85,86,87] as well as studies made after 2000 [88,89,90,91,92,93]. A version, adapted by Szałachowski [94], was used in the research. The questionnaire consists of 5 items, and the answers are evaluated on a 3-point Likert scale where 0—‘almost never’, 1—‘sometimes’, and 2—‘almost always’. The overall score is the sum of points earned on each question. Each of the five questions is rated on a scale from 0 to 1 points, and the higher the score, the higher the level of satisfaction with the family’s functioning. The responses to individual questions yield results in five specific scales, such as (1) adaptation, understood as the use of family and outside-the-family resources to solving problems and addressing stressful and critical situations; (2) partnership, understood as joint decision-making and taking responsibility for those decisions by all members of the family; (3) growth, understood as physical and emotional self-realisation achieved through mutual support and assistance; (4) affection, relationship of mutual love and care and exhibition of love and care; and (5) resolve, understood as a commitment to dedicate to and protect time for other family members [95]. The tool has satisfactory psychometric properties. The α—Cronbach coefficients for individual scales and the overall result fall within the range of 0.78–0.72 [94].

Multidimensional Scale of Perceived Social Support (MSPSS) by Zimet et al. [96], in the Polish adaptation by Buszman and Przybyła-Basista [97], takes into account the multidimensionality of social support, considering three basic sources of support: a significant other, family and friends. The scale consists of 12 statements to which the respondent refers using a seven-point Likert scale where 1 means ‘strongly disagree’ and 7 means ‘strongly agree’. The higher the score obtained, the higher the level of social support the respondent perceives in their environment. The internal consistency coefficient was calculated for the whole scale and individual subscales, and its values were total score—0.88; significant person—0.91; family—0.87; friends—0.85 [96]. In subsequent research reports, the authors confirmed the good performance of the MSPSS scale in terms of both internal reliability and factor accuracy [97].

Socio-economic Status Questionnaire. This questionnaire was used to obtain general descriptive information such as age, gender, current relationship status, and possible children’s problems, i.e., socio-economic status of participants (SES).

### 2.3. Setting and Sample

Data collection lasted from September 2022 to September 2023. The data were collected online through e-mailing and web surveys. The questionnaire is available in thematic groups, on the Authors’ Universities’ servers and forums for parents. The questionnaire was available in Polish. To obtain SES data, participants completed a demographic survey prior to testing. Participation in the study was voluntary and participants did not receive any compensation.

### 2.4. Statistical Analysis

Statistical analyses were carried out using IBM SPSS Statistics v. 29.0. In order to establish the relationship between the quantitative/order variables, a correlation analysis was carried out using Spearman’s rho (*r_s_*) or Pearson r (*r*) coefficient. To compare two groups in terms of quantitative variables, the Mann–Whitney U (*Z*) test was used; when there were more groups compared, the Kruskal–Wallis H (*H*) test was used. As a post hoc analysis, the Dunn test was performed, taking into account the Bonferroni correction for significance level.

In the next step, structural equation modelling (SEM) was carried out using IBM AMOS v.29.0. to create an explanatory model of parental burnout measured by 4 dimensions. The maximum likelihood method was chosen as the estimation method. Standard measures of model fit to the data following Hu and Bentler [98] were used as measures: RMSEA ≤ 0.08; CFI ≥ 0.90. Modifications to the model were made by starting with the information provided on the basis of modification indices, and, in the next step, irrelevant paths in the model were progressively excluded. This approach has the advantage of extracting a subset of predictors that provide the best and most effective model for predicting parental burnout during a pandemic. The level of statistical significance throughout the study was set at α < 0.05 [99].

## 3. Results

### 3.1. Characteristics of the Socio-Economic Variables of the Study Group

The study involved 337 individuals—235 women (69.7%) and 102 men (30.3%). The average age of the study groups was *M_W_*—42.26 (*SD* = 7.32) and *M_M_*—44.5 (*SD* = 9.23). In the study group, more women compared to men had children with disabilities (χ^2^ = 6.409, *p* = 0.011). The study group also included more women living in rural areas compared to men (χ^2^ = 12.433, *p* = 0.014). The remaining statistics are presented in Table 1.

**Table 1:** Characteristics of the study group.

SES	Women		Men		Test Values Significance of Difference
	M	%/SD	M	%/SD	
Number of children	*n*	%	*n*	%	1.746
one child	92	39.1	45	44.1	
two children	111	47.2	46	45.1	
three children	25	10.6	10	9.8	
four children	7	3.0	1	1	
Place of residence (size of the locality)					12.433 *
village	50	21.3	7	6.9	
town up to 25 000 inhabitants	34	14.5	14	13.7	
town up to 50,000 inhabitants	31	13.2	21	20.6	
city up to 300 thousand inhabitants	35	14.9	20	19.6	
large city (more than 300,000 inhabitants)	85	36.2	40	39.2	
Level of education					0.774
primary	1	0.4	00	0	
vocational	20	8.5	8	7.8	
secondary	36	15.3	18	17.6	
higher	108	46	45	44.1	
higher and still studying	70	29.8	31	30.4	
Years of schooling	18.37	5.052	17.35	4.821	1.729
Marital status					0.128
in marriage/relationship	180	76.6	75	73.5	
single	19	8.1	14	13.7	
divorced	34	14.5	10	9.8	
widow/widower	0	0	0	0	
in separation	2	0.9	3	2.9	
Being in a relationship					0.22
yes	206	87.7	90	88.2	
no	29	12.3	12	11.8	
Relationship length	16.33	8.0	16.08	9.36	0.234
Satisfaction of relationship assessment					1.761
low	35	17	11	12.1	
average	64	31.3	34	37.4	
high	107	51.9	46	50.1	
Financial status					2.258
very bad	1	0.4	1	1	
bad	11	4.7	2	2.0	
average	121	51.5	57	55.9	
good	67	28.5	26	25.5	
very good	35	14.9	16	15.7	
Health status					4.485
very bad	2	0.9	0	0	
bad	7	3.0	3	2.9	
average	47	20	14	13.7	
good	139	59.1	60	58.8	
very good	40	17	25	24.5	
Having a child with disability					6.409 **
yes	27	11.5	3	2.9	
no	208	88.5	99	97.1	
Having a child with chronic illness					1.056
yes	13	5.5	3	2.9	
no	222	94.5	99	97.1	
Having a child with mental illness					1.797
yes	19	8.1	13	12.7	
no	216	91.9	89	87.3	
Having a child with behavioural problems					2.836
severe	11	4.7	1	1	
medium	113	48.1	51	50	
passing	111	47.2	50	49	
Having a child with learning difficulties					2.503
severe	10	4.3	1	1	
medium	84	35.7	36	35.3	
passing	141	60	65	63.7	

* *p* < 0.05; ** *p* < 0.01.

### 3.2. Variables Distributions

An analysis using the Kolmogorov–Smirnov test (Supplementary Materials Table S1) showed that none of the analysed variables followed a normal distribution. However, the skewness values for all variables (except for the three dimensions of parental burnout: contrast in parental self, feelings of being fed up with parental role and emotional distancing) were within the range <−2;2>, indicating that the skewness was not significant and that the distribution of the examined variable was not significantly asymmetric with respect to the mean [100].

### 3.3. SES Variables and Parental Burnout

The analysis showed that there were no statistically significant gender differences in this regard. Both women and men manifested similar levels of emotional exhaustion in parental role, contrast in parental self, feeling being fed up with the parental role, emotional distancing, and overall severity of parental burnout (Table 2). We additionally tracked the size effect (r_g_). The r_g_ values, when comparing the values of the analysed variables in the group of women and men, are positive and low (in the case of the analysis in question, they can range <0;1>, where 1 means total dominance of the first sample—all the values of the second sample are smaller than all the values of the first sample), which implies a very slight dominance of the values of the variables in the group of women studied compared to the values of the variables in the group of men studied, which resulted in the occurrence of no statistically significant differences between the groups.

**Table 2:** Descriptive statistics.

	M	SD	Women (*n* = 235)					Men (*n* = 102)					Z	r_g_
			M	SD	AR	Mdn	IQR	M	SD	AR	Mdn	IQR		
Emotional exhaustion in parental rol	11.51	10.55	11.88	11.11	170.72	8.00	15.00	10.66	9.11	165.04	8.50	12.25	−0.49	0.03
Contrast in parental self	5.49	6.83	5.68	7.12	170.14	3.00	6.00	5.04	6.13	166.37	3.50	5.00	−0.33	0.02
Feelings of being fed up with parental role	3.78	5.13	4.04	5.35	174.77	2.00	5.00	3.18	4.55	155.71	1.00	4.25	−1.68	0.09
Emotional distancing	2	2.69	2.05	2.77	169.79	1.00	3.00	1.91	2.5	167.19	1.00	3.00	−0.23	0.01
Parental burnout (total score)	22.82	23.25	23.69	24.38	171.41	16.00	24.00	20.81	20.39	163.45	13.00	26.00	−0.69	0.04

In order to test whether the age of the parents examined was related to their level of parental burnout, a Spearman’s rho correlation analysis was conducted. The analysis showed weak and negative correlations between age and emotional exhaustion in the parental role and overall levels of burnout. This result indicated that, the older the subjects were in the study, the lower the level of emotional exhaustion in the parental role and the lower the severity of overall parental burnout were. There are no associations between age and contrast in parental self, feelings of being fed up with the parental role, and emotional distancing. Analyses analogous to the above were carried out to determine the relationship between place of residence, education level, years of schooling and parental burnout. The analysis showed no statistically significant relationship between the variables. In the next step, it was tested whether the number of children was associated with the severity of parental burnout. A Spearman’s rho correlation analysis showed no significant relationship between the variables. This means that the level of parental burnout was not related to the number of children one had.

Analogous analyses were performed for the health status assessment. A weak and negative correlation was found between health status ratings and feelings of being fed up with the parental role. The higher one rated one’s health status, the lower the intensity of feelings of being fed up with the parental role. There were no associations with the other dimensions of parental burnout. Weak and negative correlations were found between the contrast in parental self, feelings of being fed up with the parental role, overall level of parental burnout, and material status. This result means that, the more positive one’s material status was, the lower the levels of contrast in parental self, feelings of being fed up with parental role and overall parental burnout. Relationship length was weakly and negatively correlated with emotional exhaustion in the parental role, feelings of being fed up with the parental role, and overall levels of parental burnout. This result indicates that longer relationship lengths correlated with lower levels of exhaustion, feelings of being fed up with the parental role, and lower overall levels of parental burnout. Relationship assessment was weakly and negatively correlated with the severity of emotional exhaustion in the parental role. This means that positively assessed relationships correlated with lower emotional exhaustion in the parental role (Table 3).

**Table 3:** Analysis of the relationship between sociodemographic variables and parental burnout.

	Emotional Exhaustion in Parental Role	Contrast in Parental Self	Feelings of Being Fed Up with Parental Role	Emotional Distancing	Parental Burnout (Total Score)
Age	−0.24 ***	−0.10	−0.10	−0.02	−0.18 ***
Place of residence	0.07	0.01	0.04	0.06	0.05
Level of education	0.04	−0.02	0.04	0.01	0.02
Years of schooling	0.04	−0.02	0.04	0.03	0.02
Relationship length	−0.26 ***	−0.08	−0.12 *	−0.02	−0.19 **
Satisfaction of relationship	−0.12 *	0.00	0.04	−0.06	−0.07
Number of children	0.02	0.00	−0.04	−0.03	0.00
Financial status	−0.11	−0.12 *	−0.15 **	−0.01	−0.11 *
Health status	−0.09	−0.08	−0.12 *	−0.02	−0.09

*** *p* < 0.001; ** *p* < 0.01; * *p* < 0.05.

Four groups were included in the comparison: married, unmarried, divorced, and separated, using the Kruskal–Wallis H test. The analysis showed that marital status did not differentiate the respondents in terms of their level of parental burnout. It was then examined whether being in a relationship differentiated the level of parental burnout. The analysis using the Mann–Whitney U showed no statistically significant differences between those in and out of relationships in terms of the severity of parental burnout. The data are presented in the Supplementary Materials Tables S2 and S3.

It was then examined (results of analyses using the Mann–Whitney U test) whether having a child with a disability, chronic illness, or mental illness (Table 4) differentiated levels of parental burnout. Parents of children with disabilities reported higher levels of feelings of being fed up with the parental role than parents of healthy children (weak effect). Parents of children with mental illness manifested higher levels (weak effects) of parental burnout on all dimensions and parental burnout as an overall result than parents of children without mental illness. Having a child with chronic illness did not differentiate parents in terms of the severity of parental burnout.

**Table 4:** Comparison of the severity of parental burnout according to having a child with a medical condition.

Dependent variable	AR	Mdn	IQR	AR	Mdn	IQR	Z	r_g_
	Having a child with a disability							
	No (*n* = 307)			Yes (*n* = 30)				
Emotional exhaustion in parental role	165.96	8.00	14.00	200.08	14.00	14.00	−1.83	0.10
Contrast in parental self	166.69	3.00	6.00	192.68	4.00	9.25	−1.41	0.08
Feelings of being fed up with parental role	165.59	2.00	5.00	203.92	4.00	6.25	−2.10 *	0.11
Emotional distancing	168.55	1.00	3.00	173.62	1.00	4.00	−0.28	0.02
Parental burnout (total score)	165.87	15.00	23.00	201.07	25.00	30.50	−1.89	0.10
	Having a child with chronic illness							
	No (*n* = 321)			Yes (*n* = 16)				
Emotional exhaustion in parental role	169.68	8.00	14.00	155.28	8.00	13.00	−0.58	0.03
Contrast in parental self	170.21	3.00	6.00	144.66	2.00	5.50	−1.03	0.06
Feelings of being fed up with parental role	170.42	2.00	5.00	140.44	1.00	3.50	−1.23	0.07
Emotional distancing	171.03	1.00	3.00	128.25	0.00	1.75	−1.79	0.10
Parental burnout (total score)	170.30	16.00	24.50	142.88	10.50	17.00	−1.10	0.06
	Having a child with mental illness							
	No (*n* = 305)			Yes (*n* = 32)				
Emotional exhaustion in parental role	162.82	8.00	13.00	227.94	17.00	15.75	−3.60 ***	0.20
Contrast in parental self	163.23	3.00	5.00	224.03	8.00	13.00	−3.38 ***	0.18
Feelings of being fed up with parental role	164.20	2.00	5.00	214.72	3.50	11.75	−2.85 **	0.16
Emotional distancing	164.77	1.00	3.00	209.33	2.00	6.00	−2.56 *	0.14
Parental burnout (total score)	163.05	14.00	23.00	225.69	32.00	42.00	−3.46 ***	0.19

*** *p* < 0.001; ** *p* < 0.01; * *p* < 0.05.

The analysis showed that the presence of learning difficulties was positively associated at a weak to moderate level with all dimensions of parental burnout and parental burnout as an overall result. Behavioural problems were positively correlated at a weak to moderate level with emotional exhaustion in parental role, contrast in parental self, feelings of being fed up with parental role and parental burnout as an overall result. The more severe the child’s learning difficulties and behavioural problems, the higher the severity of parental burnout was reported on the dimensions mentioned (Table 5).

**Table 5:** Correlations between ratings of child problems and parental burnout.

Dependent Variable	Having a Child with Behavioural Problems	Having a Child with Learning Difficulties
	r_s_	r_s_
Emotional exhaustion in parental role	0.41 ***	0.29 ***
Contrast in parental self	0.30 ***	0.33 ***
Feelings of being fed up with parental role	0.33 ***	0.29 ***
Emotional distancing	0.09	0.19 ***
Parental burnout (total score)	0.38 ***	0.32 ***

*** *p* < 0.001.

### 3.4. Correlations Between Parental Burnout and Family and Personal Resources

The analysis showed negative correlations at weak to moderate levels between positivity, presence of meaning in life, sense of meaning in life (MLQ total score), social support from friend and family, social support total score, all dimensions of family functionality (family adaptation, family partnership, family growth, family affection, family resolve and FAPGAR total score), and all dimensions of parental burnout. These results indicate that, with higher resources, levels of parental burnout were lower (Table 6).

**Table 6:** Correlations between parental and personal resources and parental burnout.

	M	SD	Emotional Exhaustion in Parental Role	Contrast in Parental Self	Feelings of Being Fed Up with Parental Role	Emotional Distancing	Parental Burnout– Total Score
			r_s_	r_s_	r_s_	r	r
Religious experience	80.88	90.0	0.02	0.06	0.01	0.02	−0.01
Religious beliefs	110.36	130.0	−0.04	−0.01	−0.01	−0.07	−0.11 *
Prayer	100.21	110.0	−0.20 ***	−0.10	−0.13 *	−0.18 **	−0.21 ***
Interest in religious issues	80.76	90.0	−0.08	−0.05	−0.04	−0.10	−0.11 *
Cult	90.04	90.0	−0.07	0.02	0.00	−0.03	−0.08
Centrality of religiosity (total score)	480.26	520.0	−0.08	−0.01	−0.04	−0.08	−0.12 *
Positivity	310.51	330.0	−0.21 ***	−0.31 ***	−0.28 ***	−0.25 ***	−0.30 ***
Presence of meaning of life	260.82	290.0	−0.27 ***	−0.31 ***	−0.27 ***	−0.31 ***	−0.34 ***
Search for meaning of life	180.80	190.0	0.13 *	0.11 *	0.14 *	0.07	0.14 **
Sense of meaning of life (total score)	450.62	460.0	−0.13 *	−0.15 **	−0.12 *	−0.20 ***	−0.17 **
Social support—friends	200.94	230.0	−0.14 *	−0.17 **	−0.12 *	−0.14 **	−0.14 **
Social support—family	200.91	230.0	−0.30 ***	−0.30 ***	−0.30 ***	−0.28 ***	−0.34 ***
Social support—significant other	210.62	240.0	0.00	−0.07	−0.07	−0.11 *	−0.11
Social support (total score	630.47	680.0	−0.17 **	−0.21 ***	−0.17 ***	−0.21 ***	−0.24 ***
Family adaptation	10.64	20.0	−0.18 **	−0.15 **	−0.16 **	−0.24 ***	−0.26 ***
Family partnership	10.45	20.0	−0.29 ***	−0.23 ***	−0.22 ***	−0.23 ***	−0.32 ***
Family growth	10.55	20.0	−0.23 ***	−0.22 ***	−0.17 **	−0.25 ***	−0.27 ***
Family affection	10.35	10.0	−0.44 ***	−0.37 ***	−0.39 ***	−0.31 ***	−0.45 ***
Family resolve	10.45	20.0	−0.33 ***	−0.27 ***	−0.24 ***	−0.27 ***	−0.33 ***
Family functionality (total score)	70.43	80.0	−0.40 ***	−0.34 ***	−0.33 ***	−0.34 ***	−0.42 ***

*** *p* < 0.001; ** *p* < 0.01; * *p* < 0.05.

Among the dimensions associated with religiosity, weak and negative associations were found between religious beliefs, interest in religious issues, and intensity of religiosity centrality and overall level of parental burnout, prayer, as well as all dimensions of parental burnout except for the contrast in parental self. When religiosity levels were higher on these dimensions, levels of parental burnout were lower.

Searching for meaning in life was positively and weakly related to the severity of emotional exhaustion in the parental role, contrast in parental self, feelings of being fed up with the parental role, and overall level of parental burnout. Higher levels of searching for a sense of meaning in life were associated with higher levels of parental burnout.

Social support from a significant other was only weakly and negatively correlated with parental emotional distancing, indicating that, with higher support from a significant other, levels of parental emotional distancing were lower.

These data were also analysed either by using a regression models. The results confirmed the applicability of the SEM analysis (Supplementary Materials Table S4, Charts S1 and S2).

### 3.5. Multilevel Structural Equation Modelling: An Explanatory Model of Parental Burnout

A comprehensive model was created to explain parental burnout based on the analyses conducted previously. Those variables that were significantly related to the overall level of parental burnout among the sociodemographic variables and all variables related to family and personal resources were included in the model:

(a) Sociodemographic variables significantly associated/differentially related to the level of parental burnout: age, relationship length, child mental illness, assessment of material status, having a child with behavioural problems and learning difficulties.
(b) Family resources: adaptation, affection, growth, partnership and family resolve.
(c) Personal resources: perceived social support, meaning of life, religiosity, and positivity.

**Figure 3:**
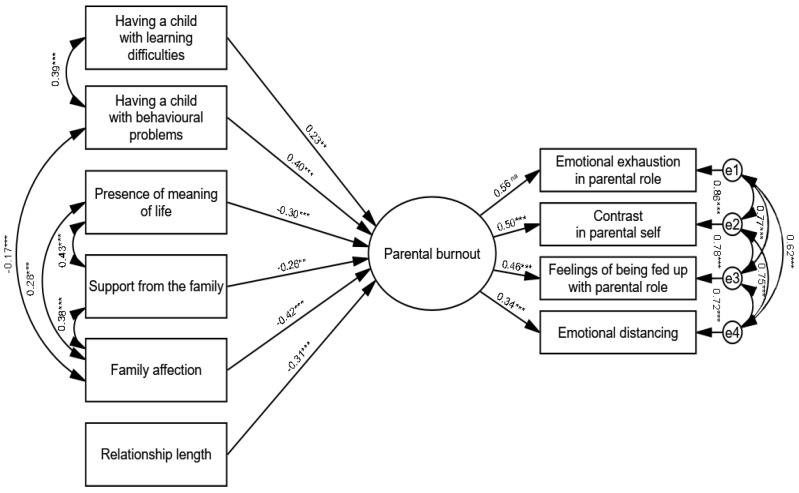
Structural equation model of parental burnout and protective/risk factors (standardised solution, n = 337). Note. χ^2^(25) = 72.46; *p* < 0.001; CFI = 0.966; GFI = 0.956; RMSEA = 0.080; 90% CI (0.059–0.088); *** *p* < 0.001; ** *p* < 0.01.

Parental burnout was included in the model as a latent variable consisting of four dimensions of burnout: emotional exhaustion in the parental role, contrast in parental self, feeling tired of the parenting role, and emotional distancing. The remaining variables were included in the model as observable variables. The maximum likelihood method was chosen as the estimation method. Based on modification indices, additional relationships between variables were included in the model. The model originally analysed (Supplementary Materials Figure S1) was not a good enough fit to the data, and therefore underwent further modifications [101]. Finally, the model included six predictors of parental burnout: learning difficulties, behavioural problems, presence of meaning in life, social support from the family, family affection, and relationship length. The model had a satisfactory fit to the data—χ^2^(25) = 72.46; *p* < 0.001; CFI = 0.966; GFI = 0.956; RMSEA = 0.080; 90% CI (0.059–0.088). In conclusion, the modified model had significant path coefficients and could be considered satisfactory in light of the fit indices considered. The standardised regression coefficients for the model are presented in Figure 3. Additional information is provided in the Supplementary Materials (Table S5).

An analysis of the results showed a positive association between the presence of behavioural problems (β = 0.40; *p* < 0.001), learning difficulties (β = 0.23; *p* = 0.007), and parental burnout, and negative associations between the presence of a meaning in life (β = −0.30; *p* < 0.001), social support from the family (β = −0.26; *p* = 0.005), family affection (β = −0.42; *p* < 0.001), and relationship length (β = −0.31; *p* < 0.001) with parental burnout. The occurrence of learning and child behavioural problems exacerbate parental burnout, whereas the other factors represent resources to reduce parental burnout. The path analysis supported the original four-factor model of parental burnout.

### 3.6. BR^2^ Index

**Figure 4:**
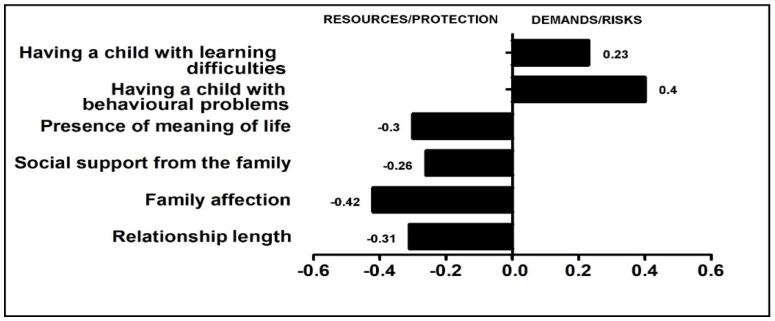
Risk and protective factors of parental burnout (weights given to predictors).

The literature presenting the results of studies compiled using SEM focuses on details, e.g., the values of path coefficients, but does not always specify the status of a specific estimator in relation to the model as a whole or its relevant substructures. This is why the next step was to determine the weights for the predictors of parental burnout according to the values of the standardised regression coefficient on the basis of the resulting model. On this basis, the BR^2^ index was calculated by averaging the values of the predictors multiplied by their weights (Figure 4). This provides a clear description of the importance of the analysed predictors. Additional information is provided in the Supplementary Materials (Supplementary Materials Chart S3).

## 4. Discussion

Confronting stressful moments, such as the COVID-19 pandemic, required the activation (or creation) of family resilience processes which, despite the crisis, created an opportunity to develop creative solutions, new dynamics in problem solving, and states of perseverance to help get through difficult experiences [102,103]. Our study adds a number of reflections to the existing literature. They mainly concern specific coupling mechanisms connecting the studied variables into loops and the importance of the group of factors.

**Affection in the family as a resource**. It is important to understand the family affection construct proposed by Smilkstein [87], which includes a relationship of mutual love and caring and the display of love and caring, which has been shown to be the most important protective factor against parental burnout. This is consistent with Epstein’s approach in the McMaster Model of Family Functioning. According to Epstein et al. [104], affective reactivity refers to the family’s ability to respond with adequate emotions to the family member’s situation (family community sadness in response to the personal sadness of a member of the family, family sympathy in the case of an individual’s unpleasant experiences, or shared happiness in family life). We can also refer here to the statements of Floyd and Morman [105] indicating that non-verbal feelings refer to physical signs of affection (e.g., gestures of affection) while verbal feelings, such as expressed oral statements of adoration (e.g., ‘I love you’), and supportive feelings include assistance in problem solving. Affectionate communication refers to how affection is expressed along nonverbal, verbal, and supportive modes of communication [106]. The aforementioned factor is close to relationship satisfaction according to Lewis and Spanier [107], and it is built over time through the exchanging of emotional messages. Our results suggest that most of the behaviours identified in Lewis and Spanier’s [107,108] model are highly relevant in a critical situation.

**Social support**. The second predictor was found to be support received within the family. In accordance with the concept of Zimet [96], which argues that perceived social support is an essential interpersonal resource for coping with stress, we focused on the perceived availability of the named support. **Social support in positive loops**. The model confirms that social support, obtained within the family system, not only reduced the risk of parental burnout but also had a strengthening effect on the sense of meaning in life and family affection of the examined individuals. The results suggest that the presence of social support within the family is a path through which not only parental burnout symptoms are alleviated but also bonding experiences are strengthened. The ability to perceive the need for and provide support may serve as an intervention objective to reduce the negative psychological effects of crisis situations [45,109,110,111].

The model or pattern of **family adaptation** identified in this study, defined by family support and family affection (they remain in a positive loop—Figure 3), results from situational events in the family environment, and, in order to adapt and solve the problem, the family must take into account the communication of emotions and the possibility of activating supportive behaviours. It seems that the two dimensions of positive affection and support are intertwined in a specific construct, i.e., functional emotional communication, through which family members know that they are accepted by others and can count on their help. This is therefore, in principle, strongly linked to emotional regulation, as has been argued in the past and present literature [11,14,112,113,114,115].

**Meaning of life as a resource in coping with a difficult situation**. Steger et al. [73] defined meaning in life as the degree to which people understand, perceive meaning, and give meaning to their lives, as well as the degree to which they perceive themselves as having a purpose or mission in life. Frankl [71] argued that meaning is an intrapersonal resource that can be drawn on to maintain well-being and adaptive functioning. Research shows that a life to which the individual does not attach meaning makes them susceptible to experiencing problems and underutilisation of skills and abilities due to lacking overriding reasons fundamental to making the effort necessary to go beyond current affairs. It looks like our research correlates between the felt meaning of life and one of the three components distinguished in the literature [71,116,117], i.e., the sense felt via relationships with the others. The most relevant research to date has focused on examining the impact of meaning in life on happiness, aiming to improve people’s experience of happiness in everyday life [118]. Research points to its relationship with well-being and links its absence to personal crisis [67,118,119,120,121]. Our research identified the felt meaning of life to be a predictor of parental burnout, thus confirming its resource-based nature. Therefore, if we accept an assumption that parental burnout is a kind of cognitive–emotional–motivational defect, then having a meaning in life, probably, contributed a specific value, thanks to which this ‘defect’ has been neutralised/blocked and the stresses experienced (pandemic) did not cause burnout, as the parental action constituted a fragment of a wider, meaningful context.

**Relationship length and parental resilience**. The longer the marriage length of the study participants, the lower the severity of parental burnout. Relationship longevity thus appears to be a resource [122]. Meta-analyses indicate that intimate relationship quality and stability is a relatively stable construct, with lower stability occurring in young adulthood and in the first years after the start of the relationship [48]. This can be related to constructive commitment (intimacy) and mature communication achieved over time [123,124,125,126], which are seen in long-term relationships and act as effective protective factors [127,128,129].

**SES in the BR^2^ model**. Although Mikolajczak and Roskam [14] in their earlier work do not recommend the inclusion of such variables in the BR^2^ model, later on, some SES factors, especially those related to children’s health, gained, according to them, the status of loadings [11,37]. In our study, they also turned out to be predictors of parental burnout during the COVID-19 pandemic. These included children’s behaviour problems and learning difficulties. It should be remembered that the BR^2^ model was designed and validated before the pandemic. It seems that the unprecedented situation of the pandemic may have changed the meaning of individual parameters somewhat (and so, for example, being constantly at home with a child demonstrating behavioural disorders or having to help a child with learning difficulties may have exacerbated parental stress). The experiences of being at home almost continuously and having to help children in school on an unprecedented scale may also have changed previous sources of stress [130,131]. Such a situation can turn a relationship with a child into a difficult one that is based on very different rules than were in place before the pandemic. We should keep in mind that the constraints of COVID-19 required families to isolate themselves from their social networks. Lock-down may have exacerbated children’s problems, placing families in a difficult situation [132,133], creating feelings of isolation and loneliness, and putting additional pressure on family relationships. In the case of parents experiencing children’s physical, medical, or mental health needs, the loss of access to social support for the purpose of helping with parental responsibilities may have exacerbated feelings of parental stress and burnout [15,134,135]. It is also reasonable to assume that the problems exhibited by the children were probably already present and that the pandemic prevented or limited the families’ use of professional help (from the school and from a doctor, psychologist, or therapist), forcing them to deal with the children’s problems on their own, which exacerbated both the children’s and the parents’ difficulties [8,136,137,138]. As a result, vicarious traumatisation may have occurred in parents [139,140], occurring in the form of parental burnout. This type of traumatisation was described as a pandemic effect during the 2014–15 Sierra Leone Ebola outbreak [141].

**Provision of external resources**. The response to the pandemic exposed vulnerabilities in system preparedness [142]. It seems important, especially in critical situations, to ensure access to sources of professional help in dealing with children’s problems, as these types of situations have been shown to be the main burdens [143,144]. This is primarily the ability to access psychological support, educational support, and medical care. It seems that, during the analysed period of the pandemic, it was here that the most serious deficiencies and even a crisis of access were noted [145]. The marginalisation of public health and the inadequacies of health promotion, disease prevention, and healthcare contributed to the severity of the health catastrophe during the pandemic period [146], which may also have affected the problems experienced by families in regard to caring for young patients.

**Figure 5:**
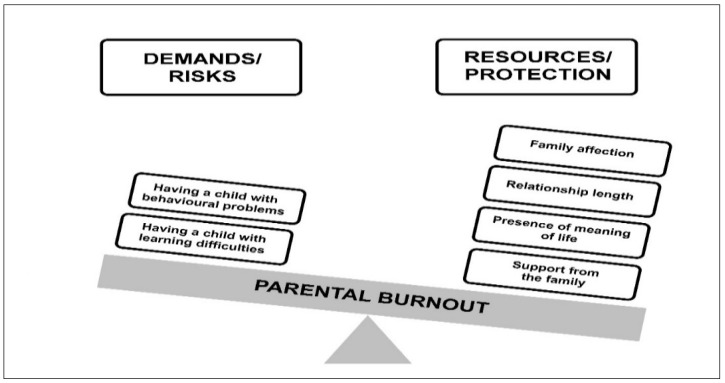
Pandemic BR^2^ model.

The present findings are instructive in both theory and practice. On a theoretical level, our results complement and strengthen the BR^2^ model:

(1) The analysis of the results confirmed the validity of the assumptions of the main hypothesis (H1) but only in relation to some exogenous variables. Analyses suggest that predictors of parental burnout appeared to be children’s behavioural problems and learning difficulties, the presence of a meaning in life, support coming from the family, family affection, and relationship length. The presence of children’s learning difficulties and behavioural problems are the most important risk factors and aggravate parental burnout, and the other factors are the main protective resources that allow parental burnout to decrease.
(2) The hypothesis (H2) that an imbalance was induced in favour of burden in the performance of parental roles during the COVID-19 pandemic should be rejected. Adaptation of the data to the BR^2^ model showed that there was no harmful imbalance between demands and resources during the pandemic. Yes, there was an imbalance, but we indicated that relatively more family resource factors than strain factors were at work during this period (Figure 5). It is worth recalling the mean parental burnout score that was obtained (*M* = 22.82; *M_w_*—23.69, *M_m_* = 20.81) and relating it to the score of 74.6 (95% confidence interval (CI) = [69.48–79.68]) identified by Brianda et al. [147] as an indicator of the clinical severity of parental burnout. This means that the level of burnout signalled by our study participants did not exceed the clinical threshold (which is probably a result of the demonstrated lack of chronic imbalance of resources and demands).

(3) ‘In accordance with the old adage that there is nothing more practical than a good theory’ [14] (p. 8), the study has been successfully operationalised—the model, derived from the path analysis, is a good fit, confirms the construct of parental burnout theory (we verified that the expected four-factor structure was confirmed also on our sample), and also demonstrates the applicability of BR^2^ theory in crisis conditions. It is worth emphasising a practical aspect of the standardised estimators in the operationalised BR^2^ model: including them in the <−1;1> interval, and thus reducing them to a common denominator, facilitates the analysis, as they can be directly compared with each other—this is especially useful when the distributions of raw scores for the index variables were based on different types of interval scales. This confirms the authors’ view that the model has good operational properties.
(4) Although our findings show that problems associated with parental burnout show more variability than expected, our results indicate that crisis-based parental burnout is associated with both micro-, meso-, and exosystemic characteristics. Mesosystemic factors—family affection and relationship length—appeared to be the strongest protective factor for pandemic parental burnout, followed by microsystemic factors—presence of a sense of life and exosystemic social support—obtained from the family. However, the most important risk factor for parental burnout turned out to be exosystemic factors—the behavioural problems of the children noted at the time and the learning problems (so far, these were probably largely dealt with by the school and doctors; in an era of limited availability of support services, families largely had to cope with them on their own).

**Limitations and Future Research**. As is the case with regression analyses, it should be borne in mind that, in reality, the relationships between variables may be non-linear and may be linearly related for reasons not of causality but of covariance (correlation). However, the path analysis model is far superior to regression analysis and is a more appropriate method for analysing causal relationships. The results are also limited by the fact that all the data are self-reported and that it is desirable to confirm them in research using other indices. The other limitation is related to the need to generalise results to the national cultural context in which the relationship is examined. The basic reservation regarding online research is also concerned with their representativeness (coverage error). The study used a retrospective self-report pretest. Due to the nature of a pandemic, where changes in circumstances come about quickly and unexpectedly, the use of traditional pre-post experimental designs is not possible. While there are limitations to consider, research on the validity of retrospective pretests suggest that they are as accurate, or more so, as traditional pretest measures [102]. It is suggested that future research should be longitudinal in nature. This should help us to look more closely at the factors (and their nature, e.g., whether the demonstrated state of ’beneficial imbalance’ is sustainable and significantly beneficial in the long term) associated with parental burnout based on BR^2^ in order to develop intervention strategies. These findings not only highlight the importance of the parameters of the model but may also point to directions for interventions targeting different perceptions of COVID-19 in order to reduce stress caused by the crisis situation.

## 5. Conclusions

‘Let the system do its job and families handle the rest’. In order to be really useful, any theory should provide clear directions for intervention [14]. It can be concluded, considering the maximum abbreviation in the area of practical application, that the simplest but, at the same time, most effective help for parents in crisis may be (in addition to psychological support in positive emotional exchange, if they need it) the effective provision of specialised help for children with functioning problems, with the result that the risk of an unfavourable balance between demands and resources is reduced [148].

**Practical implications**. Practice implications suggest that governmental or community support, as well as ensuring access to professional psychiatric and psychological assistance in cases of emergency and/or exacerbation of children’s problems (emergency support system), can alleviate parenting stress and positively impact the well-being and functioning of parents in pandemic and post-pandemic eras. The COVID-19 pandemic has shown the importance of investing in healthcare infrastructures [149].

It is not a matter of if other pandemics will occur but a matter of when. ‘The COVID-19 pandemic once again reminds us of the old saying attributed to Winston Churchill: a crisis should never be allowed to go to waste’ ([150], p. 1487). It is impossible to predict when the next pandemic will occur, as they are random events, but it is probable (URL: https://hsph.harvard.edu/news/next-pandemic-not-if-but-when/ [accessed on 26 May 2024]). Therefore, let us learn from this research, because it seems that, today, no one doubts that more pandemics will occur.

## Data Availability

The data that support the findings of this study are openly available in Mendeley Data at https://doi.org/10.17632/tvpwwrj6rr.1.

## Supplementary Materials

The following supporting information can be downloaded at: https://www.mdpi.com/article/10.3390/jcm14020617/s1, Table S1. Descriptive statistics with the Kolmogorov Smirnov test; Table S2. Comparison of the level of parental burnout according to the marital status of the respondents; Table S3. Comparison of levels of parental burnout according to being in a relationship; Table S4. Regression analysis results; Chart S1. Q-Q plot; Chart S2. The histogram; Chart S3. The histogram for BR^2^ index; Figure S1. Primary model; Table S5. Unstandardised, standardised, and significance levels for equation model.
