# Supplementary material for: ‘Let the System Do Its Job and Families Handle the Rest’? Protective and Risk Factors Contributing to Pandemic Crisis Parental Burnout
Source: J Clin Med. 2025 Jan 18;14(2):617. doi: 10.3390/jcm14020617 (PMC11766127; doi:10.3390/jcm14020617)
Supplement: Supplementary file 1 [file jcm-14-00617-s001.zip › jcm-3412666-supplementary.pdf]

Roman Ryszard Szałachowski, Wioletta Tuszyńska-Bogucka[*], and Jacek Bogucki

[a]*Department of Human Sciences, University of Economics and Innovation on Lublin, Projektowa 4, 20-209 Lublin, Poland,*

e-mail: wioletta.tuszynska-bogucka@wsei.lublin.pl;

I. TESTING DISTRIBUTIONS

Table S1. *Descriptive statistics with the Kolmogorov Smirnov test.*

	M	*Me*	*SD*	*Sk.*	*Kurt.*	*Min.*	*Max.*	*D*	*p*
Religious experience	8.88	9.00	3.49	-0.05	-1.11	3.00	15.00	0.10	<.001
Religious beliefs	11.36	13.00	4.09	-0.77	-0.81	3.00	15.00	0.22	<.001
Prayer	10.21	11.00	4.32	-0.42	-1.31	3.00	15.00	0.17	<.001
Interest in religious issues	8.76	9.00	3.84	-0.07	-1.16	3.00	15.00	0.12	<.001
Cult	9.04	9.00	4.38	-0.04	-1.57	3.00	15.00	0.16	<.001
Centrality of religiosity – total score (CRS)	48.26	52.00	18.27	-0.33	-1.25	15.00	75.00	0.13	<.001
Positive orientation	31.51	33.00	5.44	-1.31	2.15	8.00	40.00	0.13	<.001
Presence of meaning of life	26.82	29.00	7.19	-1.09	0.71	5.00	35.00	0.13	<.001
Search for meaning of life	18.80	19.00	6.84	-0.04	-0.48	5.00	35.00	0.05	.034
Sense of meaning of life – total score (MLQ)	45.62	46.00	8.45	-0.64	1.52	16.00	64.00	0.09	<.001
Social support - friends	20.94	23.00	6.64	-0.99	0.05	4.00	28.00	0.18	<.001
Social support - family	20.91	23.00	6.54	-1.03	0.07	4.00	28.00	0.18	<.001
Social support - significant other	21.62	24.00	6.38	-0.99	0.08	4.00	28.00	0.16	<.001
Social support – total score (MSPSS)	63.47	68.00	16.39	-0.99	0.34	12.00	84.00	0.13	<.001
Family adaptation	1.64	2.00	0.62	-1.53	1.14	0.00	2.00	0.44	<.001
Family partnership	1.45	2.00	0.65	-0.77	-0.47	0.00	2.00	0.34	<.001
Family growth	1.55	2.00	0.67	-1.20	0.15	0.00	2.00	0.40	<.001
Family affection	1.35	1.00	0.64	-0.48	-0.68	0.00	2.00	0.29	<.001
Family resolve	1.45	2.00	0.60	-0.58	-0.58	0.00	2.00	0.33	<.001

Family functionality – total score (FAPGAR)	7.43	8.00	2.49	-1.13	0.80	0.00	10.00	0.18	<.001
Emotional exhaustion in parental role	11.52	8.00	10.55	1.22	1.35	0.00	52.00	0.17	<.001
Contrast in parental self	5.49	3.00	6.84	2.01	4.14	0.00	35.00	0.21	<.001
Feelings of being fed up with parental role	3.79	2.00	5.13	2.01	4.26	0.00	26.00	0.23	<.001
Emotional distancing	2.01	1.00	2.70	2.05	5.41	0.00	17.00	0.23	<.001
Parental burnout – total score (PBA)	22.82	15.00	23.26	1.77	3.49	0.00	126.00	0.16	<.001

Abbreviations:
Kurt – kurtosis
M – mean
Max – maximum score
Mdn – median
Min – minimum score
r_s – Spearman's rho correlation coefficient
SD – standard deviation
Sk – skewness
D – Kolmogorov-Smirnov test statistic
CI - confidence interval
Q-Q - the quantile-quantile

Table S2. *Comparison of the level of parental burnout according to the marital status of the respondents.*

Dependent variable		AR	*Mdn*	*IQR*	*H*(3)	η^2
Emotional exhaustion in parental role	Married (*n* = 255)	165.87	8.00	12.00	3.14	<0.01
	Single (*n* = 33)	194.91	12.00	24.50		
	Divorced (*n* = 44)	171.24	7.50	15.00		
	Separated (*n* = 5)	137.80	6.00	4.50		
Contrast in parental self	Married (*n* = 255)	168.43	3.00	6.00	0.61	<0.01
	Single (*n* = 33)	179.67	4.00	8.50		
	Divorced (*n* = 44)	166.31	2.50	6.50		
	Separated (*n* = 5)	151.30	1.00	5.00		
Feelings of being fed up with parental role	Married (*n* = 255)	167.28	2.00	4.00	4.75	<0.01
	Single (*n* = 33)	197.64	4.00	9.00		
	Divorced (*n* = 44)	163.53	1.00	5.00		
	Separated (*n* = 5)	115.80	1.00	2.00		
Emotional distancing	Married (*n* = 255)	172.14	1.00	3.00	6.07	<0.01
	Single (*n* = 33)	162.53	1.00	3.00		
	Divorced (*n* = 44)	166.89	1.00	3.00		
	Separated (*n* = 5)	70.00	0.00	0.00		
Parental burnout (total score)	Married (*n* = 255)	167.43	15.00	22.00	2.88	<0.01
	Single (*n* = 33)	190.61	27.00	39.00		
	Divorced (*n* = 44)	167.30	15.50	27.50		
	Separated (*n* = 5)	121.70	8.00	7.50		

Table S3. *Comparison of levels of parental burnout according to being in a relationship.*

Dependent variable	Being in a relationship						*Z*	r_g
	No (*n* = 41)			Yes (*n* = 296)				
	AR	*Mdn*	*IQR*	AR	*Mdn*	*IQR*		
Emotional exhaustion in parental role	169.50	9.00	21.00	168.93	8.00	13.00	-0.04	<0.01
Contrast in parental self	182.89	4.00	7.00	167.08	3.00	6.00	-0.98	0.05
Feelings of being fed up with parental role	171.83	1.00	7.50	168.61	2.00	5.00	-0.20	0.001
Emotional distancing	165.80	1.00	3.00	169.44	1.00	3.00	-0.23	0.01
Parental burnout (total score)	172.22	15.00	33.50	168.55	16.00	23.00	-0.23	0.01

II. REGRESSION ANALYSIS

A series of analyses using regression analysis were performed to avoid the erroneous decision to use the SEM method.
Rest of analysis performed based on linear regression analysis:

- VIF at acceptable level <5;
- distributions of residuals close to normal distribution (Q-Q plot and histogram);
- observations >3SD were excluded.

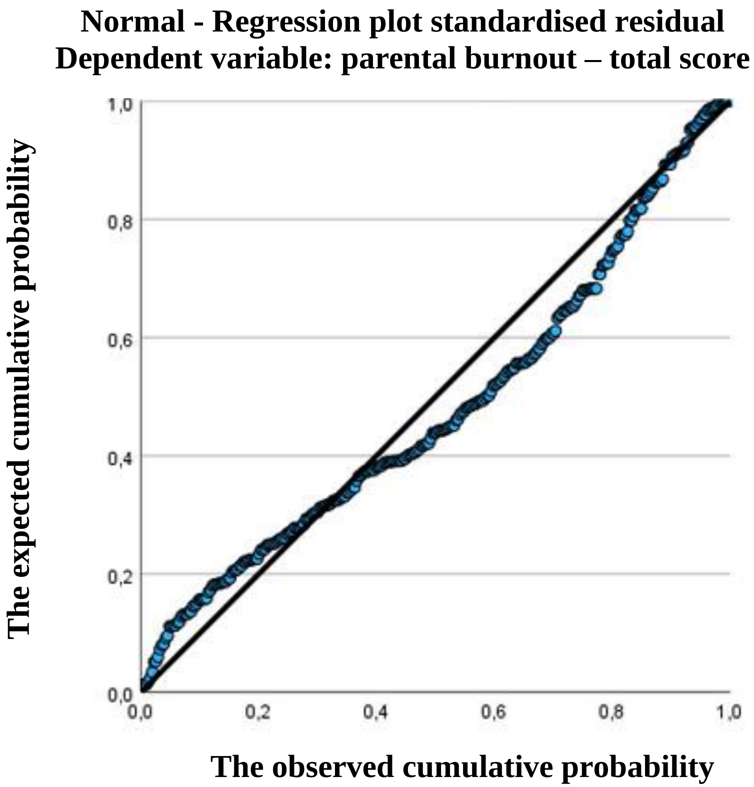

Chart S1. Q-Q plot

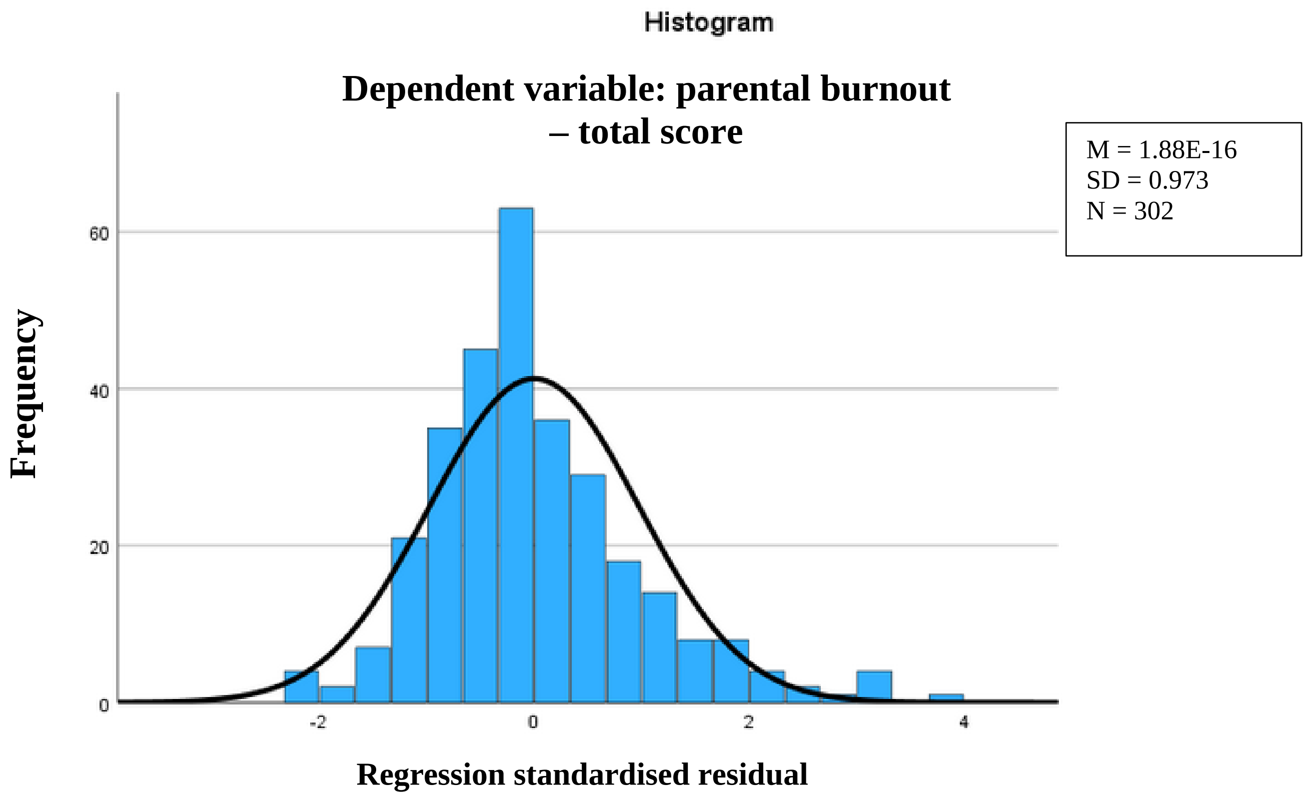

Chart S2. The histogram

Model 1 - Hierarchical regression - variables added in steps (stepwise method):

1) Sociodemographic variables -> having a child with behavioural problems and learning difficulties;
2) Family resources -> relationship lenght, family affection and resolve;
3) Personal resources -> family adaptation, affectiona nd growth, presence of meaning of life, perceived social suport from family, prayer, cult (these predictors are relevant when we consider all areas of interest).

Model 2 – backward elimination metohod

All variables are initially included in the model and, using the elimination method, those that are not significant are excluded.

Table S4. *Regression analysis results.*

	STEPWISE METOHOD						BACKWARD ELIMINATION METOHOD					
	Unstandardised		Standardised		CI 95% B		Unstandardised		Standardised		CI 95% B	
	B	SE	Beta	t	UL	LL	B	SE	Beta	t	UL	LL
(Constant)	46.38	9.60		4.83	27.49	65.28	52.47	4.32		12.15***	43.96	60.97
Age	0.17	0.18	.07	0.93	-0.19	0.52				-		
Relationship lenght	-0.40	0.16	-.17	-2.42*	-0.72	-0.07	-0.27	0.12	-.12	-2.31*	-0.49	-0.04
Having a child with mental illness	0.57	3.54	.01	0.16	-6.39	7.53				-		
Material status	0.58	1.42	.02	0.41	-2.23	3.38				-		
Having a child with behavioural problems	4.41	2.56	.13	1.72	-0.63	9.44	5.06	1.83	.15	2.76**	1.45	8.66
Having a child with learning difficulties	6.44	1.97	.18	3.26**	2.56	10.32	7.29	1.77	.21	4.12***	3.81	10.78
Family adaptation	-4.49	2.20	-.14	-2.04*	-8.82	-0.16	-3.91	1.98	-.13	-1.97*	-7.80	-0.01
Family partnership	5.31	2.31	.18	2.30*	0.75	9.86	5.21	2.11	.18	2.47*	1.06	9.36
Family growth	1.47	2.10	.05	0.70	-2.67	5.60				-		
Family affection	-6.67	1.95	-.22	-3.42***	-10.50	-2.83	-6.04	1.77	-.20	-3.42***	-9.52	-2.56
Family resolve	-4.17	1.95	-.13	-2.14**	-8.02	-0.33	-4.66	1.83	-.15	-2.56*	-8.26	-1.07
Religious experiences	0.72	0.49	.13	1.46	-0.25	1.68	0.91	0.42	.17	2.17*	0.09	1.74
Religious beliefs	0.33	0.48	.07	0.69	-0.61	1.27				-		
Prayer	-1.89	0.45	-.42	-4.21***	-2.78	-1.01	-1.65	0.37	-.37	-4.48***	-2.37	-0.92

STEPWISE METOHOD							BACKWARD ELIMINATION METOHOD					
Interest in religious issues	0.23	0.48	.05	0.49	-0.71	1.18				-		
Cult	1.40	0.40	.31	3.49***	0.61	2.19	1.46	0.37	.33	3.90***	0.72	2.19
Positivity	-0.40	0.22	-.11	-1.80	-0.83	0.04				-		
Presence of meaning of life	-0.41	0.17	-.15	-2.48*	-0.74	-0.09	-0.57	0.14	-.22	-4.05***	-0.85	-0.30
Search of meaning of life	0.15	0.14	.06	1.10	-0.12	0.43				-		
Social support – friends	0.12	0.18	.04	0.67	-0.24	0.48				-		
Social support – family	-0.56	0.27	-.18	-2.05*	-1.10	-0.02	-0.42	0.19	-.14	-2.21*	-0.78	-0.05
Social suport – significant other	0.09	0.20	.03	0.46	-0.30	0.48				-		
Adj.R^2 = .411							Adj.R^2 = .418					

The application of the regression analysis method confirmed the selection of predictors.

III. WORK ON THE MODEL

The model originally analysed was not a good enough fit to the data - $\chi^2(224) = 82.55$; $p < .001$; CFI = .846; GFI = .810; RMSEA = 0.095; 90% CI (0.088 – 0.102), and therefore underwent further modifications. The model includes pathways that are not significantly related to parental burnout. Overall scores instead of resource dimensions are included here. Models with latent variables for resources were insufficiently well fitted to the data. So were the variants taking into account the overall parental burnout score.

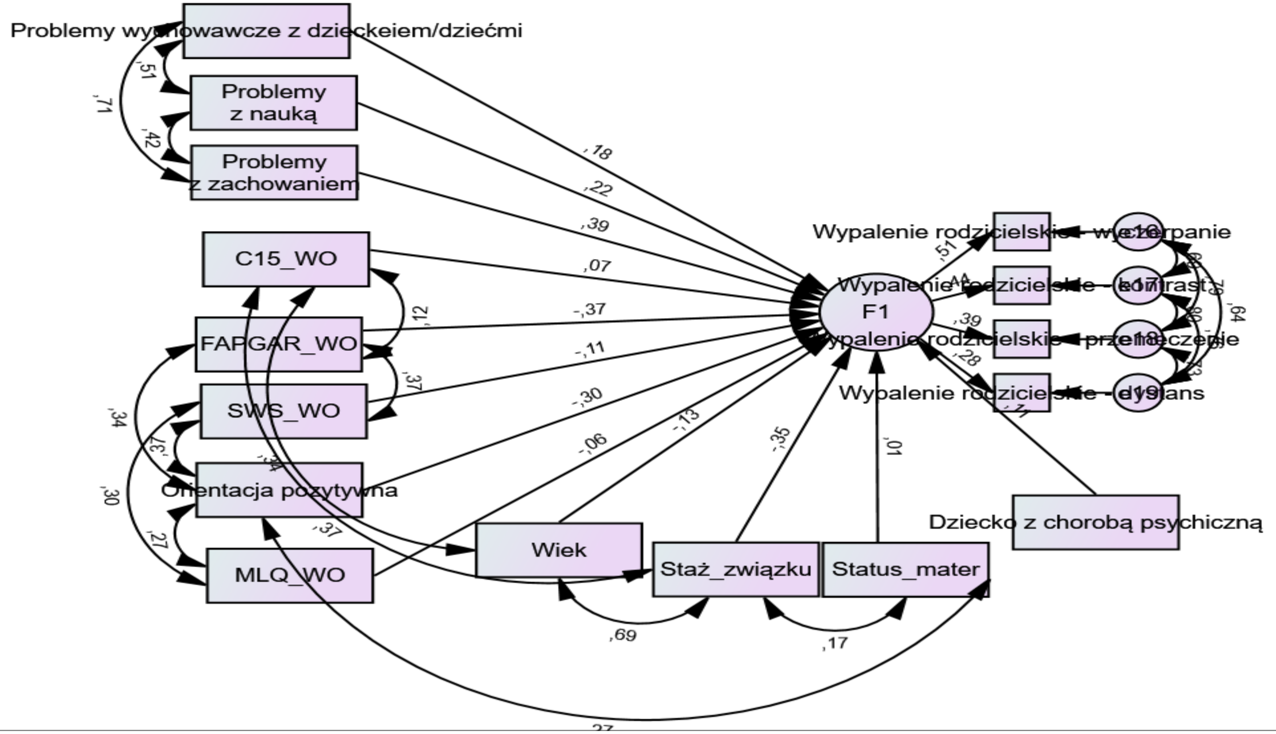

Note: $\chi^2(224) = 82.55$; $p < .001$; CFI = .846; GFI = .810; RMSEA = 0.095; 90% CI (0.088– 0.102)

Figure S1. Primary model

IV. ADDITIONAL INFORMATION ABOUT THE BR² INDEX

Table S5. *Unstandardised, standardised, and significance levels for equation model.*

	Unstandardized	*SE*	Standardized	*p*
**Measurement model estimates**				
Parental burnout -> Emotional exhaustion in parental role	1.00		.56	Na
Parental burnout -> Contrast in parental self	0.59	0.05	.50	<.001
Parental burnout -> Feelings of being fed up with parental role	0.40	0.03	.46	<.001
Parental burnout -> Emotional distancing	0.16	0.02	.34	<.001
e1	66.92	5.53		<.001
e2	33.35	2.76		<.001
e3	18.28	1.51		<.001
e4	5.94	0.49		<.001
Covariance Having a child with behavioral problems & Having a child with learning difficulties	0.12	0.02	.39	<.001
Covariance Having a child with behavioral problems & Family affection	-0.06	0.02	-.17	<.001
Covariance Presence of meaning of life & Family affection	1.35	0.29	.28	<.001
Covariance Presence of meaning of life & Support from the family	20.16	3.00	.43	<.001
Covariance Support from the family & Family affection	1.53	0.25	.38	<.001
Covariance e1 & e3	27.03	2.58	.77	<.001
Covariance e2 & e4	10.57	1.03	.75	<.001
Covariance e1 & e4	12.42	1.37	.62	<.001
Covariance e2 & e3	19.33	1.83	.78	<.001
Covariance e1 & e2	31.35	3.31	.66	<.001
Covariance e3 & e4	7.51	0.75	.72	<.001
Structural model				
Having a child with behavioral problems -> Parental burnout	4.08	0.91	.40	<.001
Having a child with learning difficulties -> Parental burnout	2.33	0.87	.23	.007
Relationship length -> Parental burnout	-0.21	0.05	-.31	<.001
Presence of meaning of life -> Parental burnout	-0.23	0.07	-.30	<.001
Support from the family -> Parental burnout	-0.23	0.08	-.26	.005

Family affection -> Parental burnout	-3.70	0.78	-.42	<.001

Note: $\chi^2(25) = 72.46$; $p < .001$; CFI = .966; GFI = .956; RMSEA = 0.080; 90% CI (0.059 – 0.088)

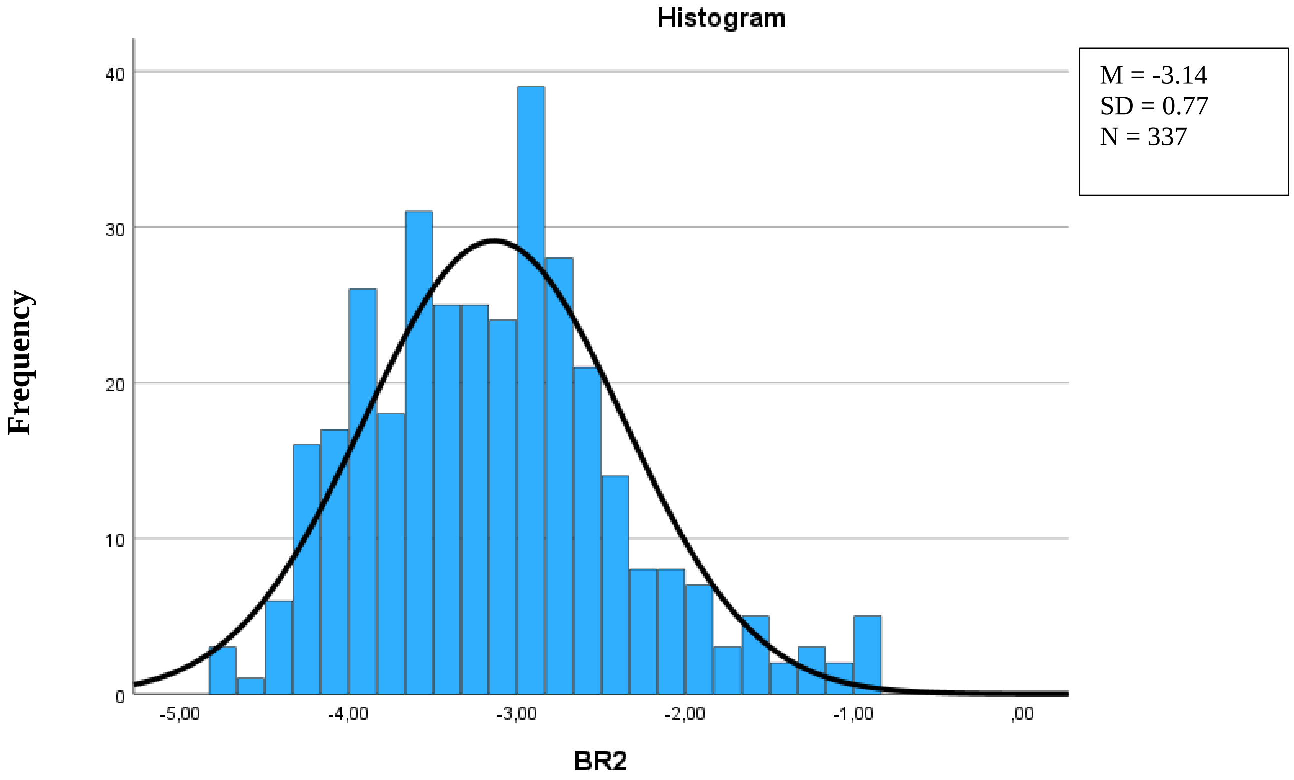

Chart S3. The histogram for BR^2 index
